# 7,8-Dihydroxy Flavone Induces Apoptosis via Upregulation of Caspase-3 in Human Hepatocarcinoma Cell 

**DOI:** 10.5152/eurasianjmed.2023.22283

**Published:** 2023-10-01

**Authors:** Gülsüm Abuşoğlu, Mukaddes İrem Durmuş, Serdar Karakurt

**Affiliations:** 1 Department of Medical Laboratory Techniques, Selçuk University Vocational School of Health Sciences Medical Services and Techniques Institution, Konya, Turkey; 2 Department of Biochemistry, Selçuk University Faculty of Science, Konya, Turkey

**Keywords:** 7, 8-DHF, apoptosis, Bax, Bcl-2, cleaved-caspase-3, HUH-7 cells

## Abstract

**Objective:**

7,8-Dihydroxyflavone, a tyrosine kinase receptor agonist, is a flavonoid that has recently gained the attention of researchers due to its anticancer properties. Nevertheless, molecular pathways of 7,8-dihydroxyflavone for hepatocarcinoma are uncertain. Our aim was to identify the impact of 7,8-dihydroxyflavone on human hepatocarcinoma.

**Material and Methods:**

Human hepatocarcinoma cell line-7 cells were used as human hepatocarcinoma cells, and 7,8-dihydroxyflavone was applied to the cells at various doses. The cytotoxic and apoptotic effects of 7,8-dihydroxyflavone were determined with Alamar Blue and flow cytometry. The properties of 7,8-dihydroxyflavone on the mRNA expressions related with Bcl-2, Bax, cleaved-caspase-3 genes, and protein expressions were determined via quantitative real-time polymerase chain reaction and western blot analysis, respectively.

**Results:**

7,8-Dihydroxyflavone-enhanced cell death in human hepatocarcinoma cell line-7 via the overexpression of cleaved-caspase-3 (*P* = .003) and decreased Bcl-2 (*P* = .038) protein levels. Furthermore, cleaved-caspase-3 mRNA overexpression (*P* = .001) markedly led to 7,8-dihydroxyflavone-induced apoptosis.

**Conclusion:**

7,8-Dihydroxyflavone could promote apoptotic cell death by modulating caspase pathways and suppressing antiapoptotic protein. These characteristics may mediate to clinical practice of 7,8-dihydroxyflavone for prevention and therapy of hepatocarcinoma.

**Main Points**

- No studies have been reported about the molecular pathways responsible for the impacts of 7,8-dihydroxyflavone (7,8-DHF) on hepatocellular carcinoma.
- The cytotoxic effects of 7,8-DHF on human hepatocarcinoma cell line-7 (HUH-7) cells were determined with Alamar Blue. The effects of 7,8-DHF on the mRNA and protein expressions were analyzed by quantitative real-time polymerase chain reaction and western blot, respectively.
- 7,8-Dihydroxyflavone-inhibited proliferation of HUH-7 cells and induced apoptosis on HUH-7 cells via caspase-dependent pathway.
- Cleaved-caspase-3 expression enhances tumor formation and is a critical step in the continuation and evolvement of cancer. 7,8-Dihydroxyflavone and associated molecules may have significant molecules for cancer therapy.

## Introduction

Hepatocellular carcinoma (HCC) is the most common diagnosis of liver cancer–related deaths. Surgery, chemo- or radiotherapy are treatment options according to the stage of the tumor. Besides that, the evolvement of promising treatment alternatives is needed for effective therapy. In recent years, many researches have been performed for clarifying anticancer molecules in natural substances.^1-4^

Phenolic molecules are abundantly present in fruits, vegetables, spices, tea, red wine, and different medicinal herbs.^5-7^ Flavonoids are sorted into different subtypes such as flavones, flavonols, flavanones, flavanonols, isoflavones, and flavanols due to the scavenging capacities of free radicals.^3^ Previous studies demonstrated antioxidant, anti-inflammatory, antimicrobial, antitumoral, or cardioprotective properties of flavonoids.^3,5-8^ The effects are primarily focused on cancers,^3,4,9^ Alzheimer’s disease (AD),^10,11^ atherosclerosis,^12,13^ and biochemical pathways.^14,15^ These compounds could affect many biological functions by scavenging free radicals, stimulating apoptosis and signal transduction pathway or inhibiting proliferation in human cancer cells.^6,15^

**Figure 1. A-D.**
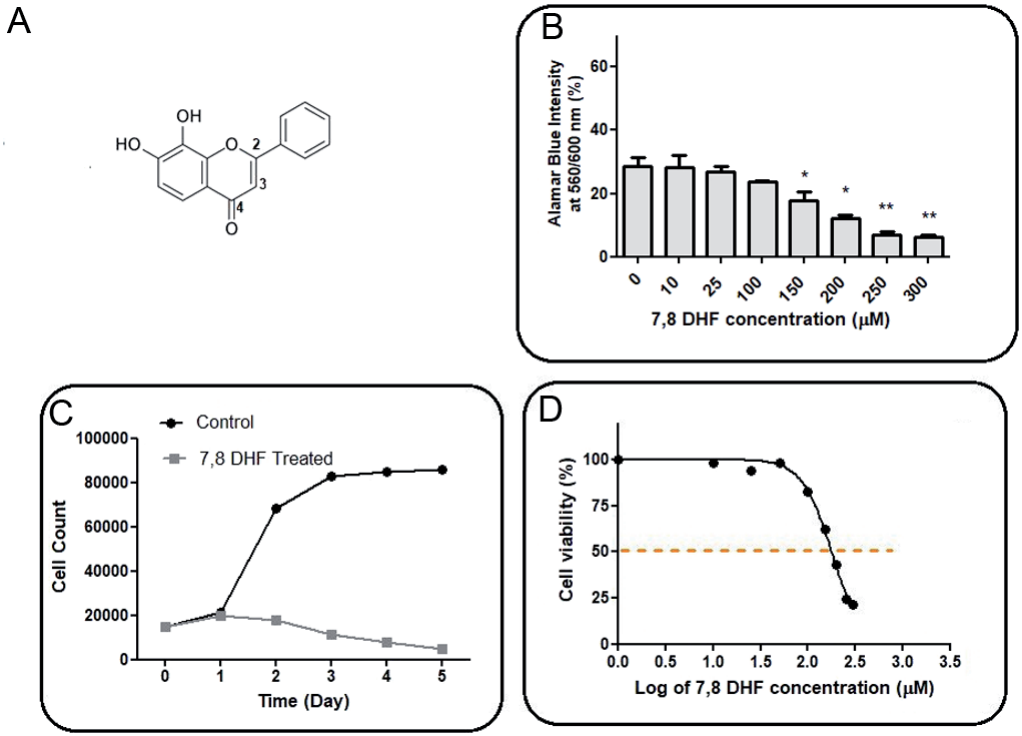
The effects of 7,8-dihydroxyflavone (7,8-DHF) on human hepatocarcinoma cell line-7 (HUH-7) cells (time- and dose-dependent manner). (A) Chemical structures of 7,8-DHF. Human hepatocarcinoma cell line-7 cells were seeded into 96-well plates, and cells were exposed to 7,8-DHF 24 hours later at the indicated dose for the indicated time periods. Effects of 7,8-DHF on viability and proliferation of HUH-7 cells; (B) viability of HUH-7 cells after treatment with various concentrations (1-300 μM) of 7,8-DHF at 48 hours measured by Alamar Blue assay at 560-620 nm; (C) the growth of HUH-7 cells, cell count vs. time; (D) IC_50_ value of 7,8-DHF on HUH-7 cells determined from sigmoidal plot of 7,8-DHF concentration (μM) vs. cell viability (%). Columns are shown as mean ± SD (^*^ *P *< .05, ^**^ *P *< .01).

Among them, 7,8-dihydroxyflavone (7,8-DHF) (Figure 1A) with anticancer effects^16^ was found in different sources such as cherries, soybeans.^15-17,18^ Also, it has neurotrophic effects. Besides that, 7,8-DHF presents antioxidant capacity by inhibiting glutamate-stimulated, 6-hydroxydopamine-stimulated dopaminergic neurotoxicity, and oxidative stress-stimulated genotoxicity.^19^ 7,8-Dihydroxyflavone has protective effects by downregulation of nuclear factor erythroid 2–related factor 2 and Heme oxygenase 1 in the liver.^17^

7,8-Dihydroxyflavone was previously associated with Alzheimer’s and Parkinson’s diseases.^10,11^ A structure–function connection study demonstrated that the catechol part in 7,8-DHF is fragile for agonistic effect; nevertheless, the catechol part is commonly metabolized by methylation, glucuronidation, sulfation in the liver, and this leads to limited oral bioavailability.^16,17^

The inhibitory properties of 7,8-DHF in various cancer cell lines have been demonstrated.^15,20^ Park et al^15^ concluded that 7,8-DHF causes cell death of leukemia cells by blocking G1 phase in the cell cycle. Lee et al demonstrated that 7,8-DHF could reorganize Sp1 activity and trigger apoptosis by modulating the cell cycle and diminishing antiapoptotic protein levels. No researchers have reported the molecular pathways responsible for these impacts on HCC. Our aim was to examine the cytotoxic and apoptotic effects of 7,8-DHF including cell viability, apoptosis, and cell cycle–related protein and gene expressions on human hepatocarcinoma cells.

## Materials and Methods

### Cell Cultures

The human hepatocarcinoma cancer cell lines, human hepatocarcinoma cell line-7 (HUH-7), were kindly provided by Department of Biological Sciences, Middle East Technical University. Ethics committee approval is no longer needed for cell culture studies. Cells were grown in Dulbecco’s modified Eagle’s medium (DMEM; Biological Industries, Cromwell, Conn, USA) containing 10% fetal bovine serum (FBS; Biological Industries, Cromwell, Conn, USA), 1% penicillin–streptomycin (Biological Industries, Cromwell, Conn, USA), and 2 mM l-glutamine (Biological Industries, Cromwell, Conn, USA) in a 37^o^C, 5% CO_2_ incubator. Approximately 85% of the passaged cells in the experiment were removed via trypsin.

### Cell Viability Assays

Human hepatocarcinoma cell line-7 cells were cultured at 1 × 10^4^ cells/mL into plates and incubated for a day. The cells were exposed to several dosages of 7,8-DHF, ranging from 1-300 μM and incubated at 37°C for 48 hours to find out the IC_50_ (Inhibitory Concentration) value. Cell viability was determined from the absorbances at 520 and 620 nm (Multiskan Go; Thermo Scientific Co., Waltham, Mass, USA) by AlamarBlue assay^21^

### Apoptosis Analysis

The apoptosis was performed by flow cytometer with Annexin V-APC (allophycocyanin) and 7-aminoactinomycin D (7-AAD) (BD Bioscience, San Jose, Calif, USA). The staining cells with Annexin V-APC and 7-AAD, viable cells [Q3; Annexin V-APC (–),7-AAD (–)], early apoptotic cells [Q4; Annexin V-APC(+)/7-AAD(–)], late-stage apoptotic cells [Q2; Annexin V-APC(+)/7-AAD(+)], and necrotic cells [Q1; Annexin V-APC(−)/7-AAD(+)] were synchronically analyzed. Following the treatment with IC_50_ of 7,8-DHF, the cells were cultured for 48 hours in an incubator and then stained with Annexin V-APC and 7-AAD for flow cytometry analysis (Acea Biosciences Inc., San Diego, Calif, USA).^22^

### Western Blot Analyses

Human hepatocarcinoma cell line-7 cells were cultured into a plate (1 × 10^5^ cells/well) and exposed with 7,8-DHF for 48 hours. Radioimmunoprecipitation assay buffer and extraction buffer (Cell Signaling Technology, Beverly, Mass, USA) was utilized in lysing cells with 1 mM of serine protease inhibitor. Protein levels were analyzed with the Bicinchoninic Acid Protein Assay method.^23^ About 25 μg of proteins from each group were electrophoretically isolated before incubation with primary antibodies [anti-Bcl-2 (1:1000), anti-Bax (1:1000), anti-cleaved-caspase-3 (1:1000), and anti-GAPDH (1:1000) antibodies (Bio-Rad Laboratories, Hercules, Calif, USA)]. Then, the blot was exposed with a secondary antibody conjugated with goat anti-mouse ImmunoglobulinG-Horseradish Peroxidase (IgG-HRP) by the western blot technique described by Laemmli^24^ and analysis of the band images was performed by imaging system (Syngene, Cambridge, UK) ImageJ v1.8.0 software.

### Quantitative Real-Time Polymerase Chain Reaction Analyses

RNA was isolated by the Trizol method.^25^ Human hepatocarcinoma cell line-7 cells were transfected with a concentration equivalent to IC_50_ to 7,8-DHF for 48 hours. The concentration and purity of the RNA were specified by evaluation of the absorbance at 260 and 280 nm via NanoDrope 1000 Spectrophotometer. cDNA (iScript cDNA Synthesis Kit; Bio-Rad) from the RNA population was synthesized. cDNA analysis was performed by reverse transcribing 1 µg of total RNA into cDNA. Bax, Bcl-2, cleaved-caspase-3, and GAPDH levels were analyzed via quantitative real-time polymerase chain reaction (qRT-PCR). The target gene expressions (2^−∆∆Ct^) were normalized to the endogenous GAPDH gene expression.^26^

### Statistical Analysis

The Statistical Package for the Social Sciences (SPSS) 21.0 (IBM SPSS Corp.; Armonk, NY, USA)) was used for statistical analysis. The Student’s *t*-test and Mann–Whitney *U* test were performed for comparison. Values are expressed as mean ± SD. Three replicates were done. *P*-values less than .05 were considered statistically significant.

## Results

### 7,8-Dihydroxyflavone Showed Cytotoxic Properties on Hepatocarcinoma Cells

Alamar Blue was conducted to find out cytotoxic activities of 7,8-DHF on HUH-7. IC_50_ was calculated by sigmoidal plot analysis using GraphPad. The cell viability in HUH-7 was performed after 7,8-DHF therapy (Figure 1B). Besides, the growth of HUH-7 cells was specified. Treatment day was admitted as day 1. 7,8-Dihydroxyflavone considerably demonstrated its effect on cell growth on day 2. Results revealed that 7,8-DHF exhibited high cytotoxicity with the IC_50_ value 177.6 μM at the 48th hour in HUH-7 (Figure 1D). In the next steps of the study, the cells were treated with the IC_50_ dose of 7.8 DHF.

### 7,8-Dihydroxyflavone Induces Apoptosis on Human Hepatocarcinoma Cell Line-7 Cells

**Figure 2. A-D.**
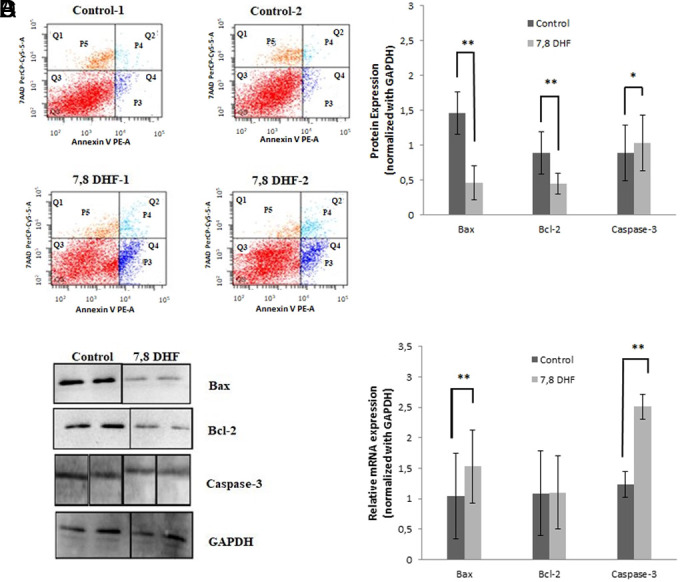
Human hepatocarcinoma cell line-7 (HUH-7) cells were treated with 177.6 μM 7,8-dihydroxyflavone (7,8-DHF) for 48 hours. (A) Effect of 7,8-DHF on apoptosis-induced cell death examined by flow cytometry following Annexin V-APC and 7-AAD staining. Western blotting was performed to analyze the Bax, Bcl-2, and caspase-3. GAPDH was used as an internal control and applied for normalization. (B) Representative immunoblot of HUH-7 cell protein in experimental control and 7,8-DHF-treated groups. (C) Analysis of band density of protein expression of the control and 7,8-DHF-treated groups. (D) Alterations in mRNA expressions were analyzed by using quantitative real-time polymerase chain reaction. Control and 7,8-DHF groups were presented with dark and light colors, respectively. Results are given as mean ± SD (statistically significant difference vs. control: ^*^ *P* < .05, ^**^ *P *< .01.

For correlation of 7,8-DHF-induced inhibition of cell growth, an alteration of apoptosis protocol was performed. Apoptosis was evaluated for 177. 6 μM of 7,8-DHF for 48 hours. After treatment, HUH-7 cells were stained to find out early and late-stage apoptotic cells (Figure 2A). 7,8-Dihydroxyflavone groups significantly exhibited early-(4.56-fold; *P* = .004) and late-stage (3-fold; *P* = .004) apoptosis in HUH-7 cells (Table 1).

### 7,8-Dihydroxyflavone Enhanced the Expressions of Proapoptotic Proteins

To further explain the regulation mechanism of 7,8-DHF on apoptosis, we analyzed the expression levels of Bax, Bcl-2, and cleaved-caspase-3 following 48 hours with 177.6 µM of 7,8-DHF treatments by western blot (Figure 2B and C). 7,8-Dihydroxyflavone incubation considerably reduced the protein expression of Bcl-2 (50.6 %; *P* = .003) and elevated levels of cleaved-caspase-3 (1.5-fold *P* = .038) in HUH-7 cells compared to controls. In addition, proapoptotic Bax protein expression was interestingly decreased (68.4 %; *P* = .004). According to these results, Bax/Bcl-2 ratios were found to be higher in the 7,8-DHF group compared to (Bax/Bcl-2: 1.64 for control; Bax/Bcl-2: 1.02 for 7,8-DHF).

### Gene Expression Analyses

Quantitative real-time polymerase chain reaction analyses demonstrated crucial changes in mRNA of Bax, Bcl-2, and cleaved-caspase-3 genes related to the apoptotic pathway, as shown in Figure 2D. In 177.6 μM 7,8-DHF-treated group, Bax (1.47-fold; *P* = .004) and cleaved-caspase-3 (2.02-fold; *P *= .001) expression levels were significantly higher in HUH-7 cells than controls. Besides, mRNA expression levels of Bcl-2 were not significantly different (*P* = .087).

## Discussion

The major molecular construction of a flavonoid includes 2 aromatic rings (A and B) attached by pyran-4-one ring C and roughly 4000 flavonoids found on earth.^3,6^ The position of the double bond in the C ring is proposed to be significant determinants for flavonoids’ physiological activities. Otherwise, hydroxyl groups at C ring and the count of hydroxyl groups at B ring play a crucial role in the activity of flavonoids. It was suggested that the multifunctional impressions of flavonoids were due to structural and functional groups.^3,6^

7,8-Dihydroxyflavone is a potent TrkB agonist in several disorders.^27,28^ 7,8-Dihydroxyflavone was notified to exhibit significant neurotrophic and antioxidant capacity.^6,28-30^ 7,8-Dihydroxyflavone was previously defined as a possible anticancer agent.^19^ Besides, the exact linkage of the anticarcinogen properties of 7,8-DHF is uncertain. Small numbers of flavonoids were presented to inhibit cell proliferation by triggering apoptosis in hepatocarcinoma cell lines.^3,4,6,9,17^ Despite the prohibitory effect of 7,8-DHF in certain kinds of carcinoma,^19,20,31^ no data were reported about hepatocarcinoma yet. According to the results of our study, 7,8-DHF seems to inhibit HUH-7 cell proliferation by increasing apoptotic cell death.

Park et al^31^ exposed U937 cells with 7,8-DHF for analysis of cell proliferation by methyl-thiazol-tetrazolium (MTT). IC_50_ dose was 70 μM in U937 cells for 72 hours. Lee et al^20^ treated oral squamous cancer cells with several concentrations of 7,8-DHF. They reported that 40 μM of 7,8-DHF inhibited cell proliferation by MTT analysis. Sim et al^19^ found IC_50_ dose of 7,8-DHF as 9.04 μM on B16F10 by MTT analysis. Choi et al^32^ demonstrated that higher concentrations (>50 μM) of 7,8-DHF reduced proliferation in preadipocyte cells by the MTT method. In our study, the IC_50_ of 7,8-DHF in HUH-7 cells was detected as 177.6 μM for 48 hours using Alamar Blue assay. IC_50_ dose differences from studies may be due to different cell lines and cell viability analyzing techniques.

7,8-Dihydroxyflavone significantly increased early (4.56-fold) and late-stage (3-fold) apoptosis cycles in HUH-7 cells. Several studies have demonstrated that 7,8-DHF-induced apoptosis on different cancer cell lines such oral squamous (early and late apoptotic cells, 19.3- and 13.44-fold for 48 hours, respectively) and leukemia cancer (early and late apoptotic cells rate 40% for 24 hours).^20,31^ In this study, apoptosis was induced in HUH-7 cells treated with IC_50_ concentration of 7,8-DHF. This study’s findings are consistent as specified in the literature. The type of cancer cells may be related to the dosage and apoptotic cell rate.

Park et al^31^ showed that 7,8-DHF triggered considerably cell death via a caspase and Bax-dependent pathway. Lee et al^20^ specified that 7,8-DHF decreased the levels of Bcl-xL, enhanced Bax, and cleaved-caspase-3 levels. Choi et al^32^ reported that 7,8-DHF promotes apoptosis in preadipocyte cells via activating proapoptotic determinants like caspase family proteins. 7,8-Dihydroxyflavone may decrease the count of fat cells in adipose tissue by inhibiting the proliferation of the preadipocyte cells. In our study, a crucial increase in cleaved-caspase-3 expression was determined in 7,8-DHF treatment in HUH-7 cells. 7,8-Dihydroxyflavone induced a 50.6% decrease in Bcl-2 protein levels (*P* = .003) and 1.5-fold elevation in cleaved-caspase-3 protein levels (*P* = .038) in HUH-7 cells. Furthermore, cleaved-caspase-3 (2.02-fold increase; *P* = .001) and Bax (1.47-fold increase;* P* = .004) mRNA levels were increased by 7,8-DHF in HUH cells. Although the protein expression levels of Bcl-2 were statistically lower, the mRNA levels were not affected.

Cancer cells can use different molecular mechanisms to suppress apoptosis, which is a programmed cell death, by various biochemical and metabolic pathways. Bcl-2 members with both proapoptotic (Bax, Bak, Bid, and Bim) and antiapoptotic (Bcl-2, Bcl-xL, and Bcl-W) situations are located in mitochondria.^33^ Thus, cancer cells may obtain resistance to apoptosis by Bcl-2 (antiapoptotic) overexpression or Bax (proapoptotic) downregulation. Ever since the expression of both Bcl-2 and Bax is regulated by the p53 tumor-suppressor gene, certain forms of human B-cell lymphoma were concluded to have Bcl-2 overexpression.^34^ Cleaved-caspase-3 is a protein that interacts with caspase-8 and caspase-9 and plays a role in apoptosis. In the intrinsic pathway, cytochrome c released from mitochondria works with caspase-9, apoptosis-activating factor 1 (Apaf-1) to produce procleaved-caspase-3 with the availability ATP (Adenosine triphosphate).^35^

This study’s findings remarked that 7,8-DHF application led to the occurrence of apoptosis. Apoptosis was associated with increased levels of cleaved-caspase-3, overexpression of Bax, and decreased Bcl-2, which play roles in the initiation and regulation of the apoptosis process.^31^ These results suggest that 7,8-DHF–induced apoptosis via caspase-dependent mechanism by disrupting the cell death on HUH-7 cells compared to controls.

Limitations, drawbacks, or shortcomings: In this study, cytotoxicity and cell death analyses were performed with adequate and reliable methods. To understand the metabolic pathways of cell death, apoptotic and antiapoptotic proteins, experiments were performed with protein and mRNA steps. Notwithstanding, further protein analyses could be performed such as p53, caspase 8-9, Apaf-1, and additional cell cycle analyses could be performed to confirm the results.

In conclusion, a large number of studies analyzing novel anticancer agents and biomarker compounds that are considered useful for cancer treatment should be concentrated particularly on cellular mechanisms such as apoptosis, expression of protein and mRNA. The cleaved-caspase-3 expression enhances tumor formation and is a critical step in the continuation and evolvement of cancer. According to these results, 7,8-DHF inhibits the proliferation of HUH-7 cells by affecting programmed cell death. These new phenomena were not already defined for 7,8-DHF, and given that 7,8-DHF and associated molecules may have significant roles as agents for cancer therapy. To the best of our knowledge, we have described the molecular mechanisms of the effect of 7,8-DHF for the first time in HUH-7 cells. 7,8-Dihydroxyflavone might be a gradually impressive suppressant or remedial substance in tumor genesis.

## Figures and Tables

**Table 1.** Early and Late Apoptotic Cell Rates of Hepatocarcinoma Cell Line Cells

HUH-7	Annexin(+)/7-AAD(+) (Q2) %	AnnexinV(+)/7-AAD(−)(Q4) %
Control	1.85 ± 0.35	3.65 ± 0.63
7,8-DHF	5.55 ± 0.21	16.65 ± 4.31
*P*	.004^*^	.004^*^

For apoptosis analyses, HUH-7 cells were treated with 177.6 µM 7,8-DHF for 48 hours, and analyses were performed by flow cytometry following Annexin V-APC and 7-AAD staining. Results showing the numbers of cells in the early stage of apoptosis (marked by positive Annexin V-APC staining; Q4) and the late stage of apoptosis (stained with 7-AAD; Q2).

^*^ *P* < .01, compared with the control or 7,8-DHF-treated group.

7,8-DHF, 7,8-dihydroxyflavone; 7-AAD, 7-aminoactinomycin D; HUH-7, hepatocarcinoma cell line.
